# Association of blood group B and of rare variants affecting immune system with multisystem inflammatory syndrome in children in an Italian cohort

**DOI:** 10.3389/fimmu.2026.1777516

**Published:** 2026-04-10

**Authors:** Luisa Ronzoni, Giada Maria Di Pietro, Angela Lombardi, Lorenzo Miano, Francesca Minoia, Lucia Baselli, Giovanni Filocamo, Veronica Torcianti, Francesco Malvestiti, Samantha Bosis, Daniele Prati, Luca Valenti

**Affiliations:** 1 Precisione Medicine Lab, Biological Resource Center and Department of Transfusion Medicine, Fondazione IRCCS Ca’ Granda Ospedale Maggiore Policlinico Milano, Milan, Italy; 2 Pediatric Emergency Department, Fondazione IRCCS Ca’ Granda Ospedale Maggiore Policlinico Milano, Milan, Italy; 3 Department of Pathophysiology and Transplantation, Università degli Studi di Milano, Milan, Italy; 4 Pediatric Immuno-Rheumatology Unit, Fondazione IRCCS Ca’ Granda Ospedale Maggiore Policlinico Milano, Milan, Italy; 5 Pneumology and Infectious Diseases Unit, Fondazione IRCCS Ca’ Granda Ospedale Maggiore Policlinico Milano, Milan, Italy

**Keywords:** COVID-19, genetics, Kawasaki disease, multisystem inflammatory syndrome in children, SARS-CoV-2 infection

## Abstract

**Background:**

Multisystem inflammatory syndrome in children (MIS-C) is an uncommon delayed complication of the severe acute respiratory syndrome coronavirus 2 (SARS-CoV-2) infection in children, whose cause remains unknown. The aim of this study was to investigate the role of genetic predisposition to COVID-19 and dysregulated inflammatory response in the development of MIS-C in Italian children.

**Methods:**

Eighteen individuals were enrolled: 12 with classical MIS-C and 6 with a Kawasaki–SARS-CoV-2-related disease (KD). The frequency distribution of the main common risk variants underpinning COVID-19 susceptibility (*ABO* tagging SNPs) and severity (five GWAS-prioritized loci) was compared between patients and children with COVID-19 without MIS-C and with adult controls (*n* = 79 and *n* = 2,848, respectively). Whole exome sequencing (WES) was performed in the MIS-C cohort to examine the frequency of rare damaging variants in a panel of 207 immune-related genes as compared to that of local controls (*n* = 266).

**Results:**

Blood group B alleles conferred an increased risk of MIS-C independently of sex, ethnicity, the presence of COVID-19, and blood group A [odds ratio (OR) 2.9; 95% confidence interval (CI) 1.04–8.5; *p* = 0.04], with a larger impact on the KD subphenotype (OR 6.8; 95% CI 1.7–35.1; *p* = 0.007). A total of 49 rare damaging variants, 4 classified as pathogenic, were prioritized in 39 immune-related genes; all patients harbored at least one variant.

**Conclusions:**

These results not only support a role of blood group B as a risk factor for MIS-C development in children with COVID-19, possibly through modulation of the coagulability and microvascular dysfunction, but also support an immune-genetic basis for this condition.

## Introduction

Multisystem inflammatory syndrome in children (MIS-C) is a rare but potentially life-threatening condition that is usually manifested in 1%–6% of infected children 2 to 6 weeks after mild or asymptomatic infection with severe acute respiratory syndrome coronavirus 2 (SARS-CoV-2) (1). Although MIS-C has become less prevalent and severe over time, racial and ethnic disparities persist (2).

According to the Centers for Disease Control and Prevention (CDC) criteria, MIS-C can be diagnosed in individuals under the age of 21 in the presence of fever for greater than 24 h, laboratory evidence of inflammation, severe illness that requires hospitalization, multiorgan involvement, evidence of SARS-CoV-2 infection or exposure to a coronavirus disease (COVID-19) case within 4 weeks prior to symptom onset, and exclusion of other possible etiologies (3–7). Although MIS-C shares features with other inflammatory disorders, such as Kawasaki disease (KD), toxic shock syndrome (TSS), macrophage activation syndrome (MAS), or hemophagocytic lymphohistiocytosis (HLH) (8, 9), and despite an improved understanding of its pathophysiology since its initial description (10), there is still much to be learned regarding the mechanisms and susceptibility that lead to disease development. A post-infectious immune dysregulation leading to a cytokine storm with increased levels of interferons (IFNs) and pro-inflammatory interleukins has been suggested as a potential mechanism (11–13). Endothelial cell activation and vascular dysfunction are other key features (14).

A genetic predisposition to dysregulated inflammatory response and cytokine storm has been proposed to explain the clinical variability in MIS-C development and severity (15, 16), and the genetic background has been demonstrated to modulate COVID-19 susceptibility and severity. The strongest signal associated with increased risks of COVID-19 morbidity and mortality has been located at chromosome 3p21.31 spanning numerous genes including *LZTFL1*. Furthermore, the *ABO*-blood group system has been associated with susceptibility to SARS-CoV-2 infection with the non-O blood group predisposing to COVID-19 infection (17–21). Similarly, genetic variants in genes involved in inborn errors of immunity (IEI) (22) that were previously associated with inflammatory disorders like HLH (23), such as *LYST*, *STXBP2*, *PRF1*, *UNC13D*, and *AP3B1* genes, as well as in immune-related genes, including *TLR3*, *TLR6*, *IL22RA2*, *IFNB1*, and *IFNA6*, have also been described in children with MIS-C (24–34) genotypes A02, B35, and C04, and have been associated with an increased risk of MIS-C (35).

Within this context, we aimed to investigate the role of genetic predisposition to MIS-C development in a cohort of Italian children, evaluating first the common risk variants previously associated with COVID-19 susceptibility and then the presence of rare damaging variants in immune-related genes.

## Materials and methods

### Study cohort

The study was conducted in a tertiary care hospital (Fondazione IRCCS Ca’ Granda Ospedale Maggiore Policlinico) in Milan, Northern Italy. All subjects ≤18 years admitted to the Pediatrics Clinic from September 2020 to March 2022 with a diagnosis of MIS-C or KD related to SARS-CoV-2 infection were enrolled. MIS-C diagnosis was established according to the CDC clinical criteria, while a KD–SARS-CoV-2 related diagnosis (KD throughout the text) was performed in all children who not only met the criteria for KD diagnosis but also had a current or recent SARS-CoV-2 infection (**Supplementary Table 1**) (3, 36, 37). The correlation with SARS-CoV-2 infection was demonstrated by being positive for SARS-CoV-2 infection within the 4 weeks prior to the onset of symptoms, or by exposure to a COVID-19 case within the 4 weeks. SARS-CoV-2 infection was established with a nasopharyngeal aspirate or swab positive for SARS-CoV-2 RNA, a positive antigen test detecting the nucleocapsid protein within the past 60 days, or a positive serology (typically IgM-negative and IgG-positive, regardless of vaccination status) (38).

Data regarding sex, age at admission, ethnicity, pre-existing chronic diseases, and chronic therapies were collected for each patient; symptoms, blood tests, the need for care in the pediatric intensive care unit (PICU), length of stay, therapies administered, and the persistence of long-term sequelae were assessed. For each patient, a blood sample was collected in an EDTA tube for genetic analysis, upon obtaining written informed consent from at least one parent.

The genetic study was approved by the Ethics Committee of the Fondazione IRCCS Ca’ Granda (FoGS, approval number 342_2020).

### Genotyping of common risk variants associated with COVID-19 susceptibility

DNA was extracted from peripheral blood via QIASymphony (Qiagen, Milan, Italy). Genotyping of common variants associated with COVID-19 susceptibility identified by genome-wide association studies (GWASs) (*LZTFL1* rs11385942 tagging chromosome 3 cluster, *OAS3* rs10735079 tagging the OAS1/2/3 locus variability, *FUT2* rs601338, *IFNAR2* rs2229207, and *DPP9* rs2109069) was carried out using commercial TaqMan SNP assays and analyzed using the SDS software v.2.3 (StepOne Plus; Applied Biosystems). The two SNPs rs657152 and rs8176746, located in the *ABO* gene, were analyzed thorough Sanger sequencing, and the results were combined to infer the ABO blood type phenotype and genotype, as previously described (17, 18, 21). The variant frequencies observed in cases were compared to that of ethnically matched controls including 79 children with COVID-19 (COVID-19 pediatric controls) and 2,848 adult blood-donor population controls (adult controls) recruited during the same period at the Fondazione Hospital and for which GWAS data were already available (21).

### Rare damaging variant detection

**Figure 1:**
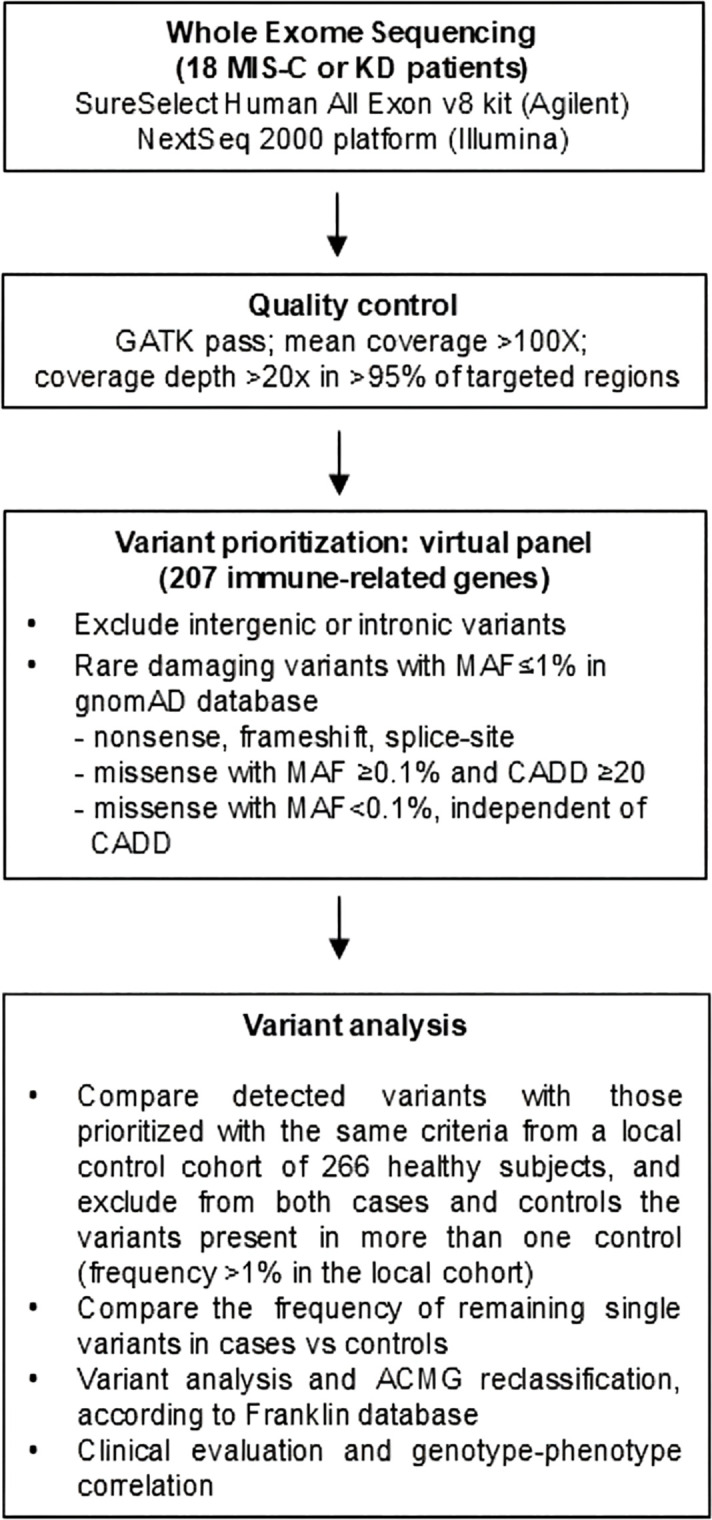
Study flowchart for rare variants detection through whole exome sequencing (WES), annotation, and secondary analysis.

DNA from peripheral blood was quantified by a Qubit 2.0 analyzer using the Qubit dsDNA BR Assay Kit (Thermo Fisher Scientific, Waltham, MA, USA). Genomic DNA libraries were enriched for whole exome sequencing (WES) by the SureSelect Human All Exon v8 kit (Agilent, Cernusco sul Naviglio, Milan, Italy) and sequenced on the NextSeq 2000 platform (Illumina, San Diego, CA). Variant calling was performed using a validate pipeline (39). Sequencing mean depth was 128 ± 29×. Each sample exhibited a percentage >96% of target regions with a coverage at 20×.We considered a virtual panel of 207 candidate genes (**Supplementary Table 2**) previously reported to be involved in MIS-C development and/or severe COVID-19 (29, 30, 32). Genes were classified into seven categories: associated with inflammatory disorders; involved in the inflammatory or IFN pathway; associated with immune dysregulation, pHLH, or primary immunodeficiency disorder (PID); or associated with COVID-19 susceptibility. Exonic non-synonymous (missense, frameshift, and nonsense) or splice-site variants were considered; among these, variants with a minor allele frequency (MAF) ≤ 0.01 according to the Genome Aggregation Database (gnomAD) were prioritized [missense variants with MAF between 0.001 and 0.01 were considered only if the CADD (Combined Annotation Dependent Depletion) score was ≥20]. Variants were then classified according to the American College of Medical Genetics (ACMG) guidelines as pathogenic (P), likely pathogenic (LP), unknown significance (VUS), likely benign (LB), and benign (B) through the Franklin platform (accessed in February 2026). We then applied the same prioritization criteria to a local control cohort of 266 subjects (local WES controls) for whom WES data generated by the same approach and analyzed by the same pipeline were available (40). Variants present in more than one control, estimated to have a frequency higher than 1% in the local population, were removed from both control and case cohorts; the frequency of the remaining variants was compared between cases and controls. The study flowchart for variant detection and prioritization is reported in **Figure 1**.

### Statistical analysis

Categorical variables were expressed as frequencies and percentages; continuous variables were expressed as mean ± standard deviation or median and interquartile range (IQR), as appropriate. Observational associations were performed by fitting data to generalized linear models; logistic models were fit to examine binary traits. In multivariable models, analyses were adjusted for relevant covariates, as detailed in the Results section. The frequencies of rare variants were compared between patients and controls using Fisher’s exact test. Statistical analysis was carried out using the JMP Pro 16.0 Statistical Analysis Software (SAS Institute, Cary, NC). *p*-values <0.05 (two tailed) were considered statistically significant.

## Results

### Study population

A total of 18 children and adolescents <18 years of age were enrolled: 12 with classical MIS-C and 6 with KD–SARS-CoV-2-related characteristics (KD). The clinical features of the overall cohort are shown in **Table 1**. The mean age at admission was 6.9 ± 4.3 years, 55% of patients were male, and the majority were Europeans (94%). Only two patients (11%) suffered from underlying chronic conditions, specifically vitiligo and febrile seizures. Hyperpyrexia, conjunctivitis, and skin rash were the most frequently reported symptoms at admission. Complications developed in 11 patients (61%), particularly cardiac dysfunction and gastroenteric involvement (in 61% and 50% of cases, respectively). The majority of patients showed increased levels of leukocytes, C-reactive protein, and ferritin (**Supplementary Table 3**). All patients received intravenous immunoglobulins and acetylsalicylic acid; treatment with corticosteroids was administered in 67% (12/18) of cases and with anakinra in 11% (2/18). All patients recovered without sequelae after a mean hospitalization of 12 days.

**Table 1:** Clinical features of the overall MIS-C cohort and after stratification according to clinical phenotype.

	Overall MIS-C (*N* = 18)	Classical MIS-C (*N* = 12)	KD (*N* = 6)	*p*-value
Age, years	6.9 ± 4.3	8.5 ± 3.5	4 ± 3.8	0.03
Sex, male	10 (55.5)	5 (41.7)	5 (83.3)	0.15
Ethnicity, European	17 (94.4)	11 (91.6)	6 (100)	1
Underlying chronic diseases, yes	2 (11.1)	0	2 (33.3)	0.09
Coinfections, yes *	7 (43.7)	4 (40)	3 (50)	1
Clinical manifestations, yes	18 (100)	12 (100)	6(100)	1
Fever	18 (100)	12 (100)	6(100)	1
Respiratory symptoms	10 (55.5)	7 (58.3)	3 (50)	1
Gastroenteric symptoms	12 (66.7)	10 (83.3)	2 (33.3)	0.11
Conjunctivitis	13 (72.2)	7 (58.3)	6 (100)	0.11
Anosmia/Ageusia	0	0	0	na
Skin rash	13 (72.2)	8 (66.7)	5 (83.3)	0.6
Seizures	1 (5.5)	0	1 (16.6)	0.33
Arthromyalgia	2 (11.1)	1 (8.3)	1 (16.6)	1
Chest pain	0	0	0	na
Complications, yes	11 (61.1)	8 (66.7)	3 (50)	0.6
Shock	1 (5.5)	1 (8.3)	0	1
Cardiac dysfunctions	11 (61.1)	8 (66.6)	3 (50)	0.6
Pneumonia/SARI/ARDS	5 (27.7)/0/0	5 (41.6)/0/0	0/0/0	0.11
Gastroenteric complications	9 (50)	7 (58.3)	2 (33.3)	0.6
Access to PICU, yes	3 (16.6)	2 (16.6)	1 (16.6)	1
Specific treatment, yes	18 (100)	12 (100)	6 (100)	na
Corticosteroids	12 (66.7)	11 (91.6)	1 (16.6)	0.02
Anakinra	2 (11.1)	2 (16.6)	0	0.5
Intravenous immunoglobulins	18 (100)	12 (100)	6 (100)	na
Sequelae, yes	0	0	0	na
Death, yes	0	0	0	na
Length of stay, days	12 [8.7–14.7]	14 [10.5–17.7]	8.5 [6.5–9.7]	0.009

Data are shown as *N* (%), or mean ± standard deviation or median [IQR], as appropriate. SARI: severe acute respiratory illness; ARDS: acute respiratory distress syndrome; PICU: pediatric intensive care unit.

* Available for 16 patients, 10 with MIS-C and 6 with KD phenotype. *p*-values were calculated among pairs through Kruskal–Wallis test for continuous variables (non-normality assumed) and Fisher test for categorical variables.

After stratification according to clinical subphenotypes, the mean age at admission was higher in the MIS-C subgroup (8.5 ± 3.5 vs. 4 ± 3.8 years, *p* = 0.03) as well as the median levels of ferritin and alanine aminotransferase (ALT) (*p* = 0.001 and *p* = 0.03, respectively). The length of stay was higher in the MIS-C subgroup (*p* = 0.009), which more frequently required corticosteroids, whereas no significant differences were observed concerning the rate of complications (**Table 1** and **Supplementary Table 3**).

### Impact of common genetic variation associated with COVID-19 susceptibility

We first analyzed ABO blood type distribution, which is the main genetic risk factor for SARS-CoV-2 infection susceptibility. In relation to this, we found that blood group B was more frequent in MIS-C compared to COVID-19 pediatric controls or adult controls [odds ratio (OR) 3.8; 95% confidence interval (CI) 1.2–12.9; *p* = 0.03, and OR 4; 95% CI 1.5–10.8; *p* = 0.01, respectively] (**Table 2A**). Specifically, the presence of the B allele conferred a 2.8-fold increased risk for MIS-C development compared to COVID-19 pediatric controls and 3.8-fold increased risk compared to adult controls, independently of sex and ethnicity (OR 2.8; 95% CI 1.03–7.6; *p* = 0.04 and OR 3.8; 95% CI 1.5–8.5; *p* = 0.002, respectively) (**Table 2B**). To further dissect the impact of the B allele on MIS-C development, we compared the MIS-C cohort to the controls overall (COVID-19 pediatric controls plus adult controls, *n* = 2927). Overall, the presence of the B allele conferred a 2.9-fold increased risk, independently of sex, ethnicity, and the presence of COVID-19 and the A allele, considered a major risk factor for COVID-19 susceptibility (OR 2.9; 95% CI 1.04–8.5; *p* = 0.04) (**Table 2C**). To an exploratory aim, we evaluated the impact of the B allele on subphenotypes (classical MIS-C or KD) development. The presence of the B allele tended to confer a higher risk for KD phenotype (OR 5.8; 95% CI 0.99–56.1; *p* = 0.07) (**Supplementary Table 4A**). The trend was confirmed comparing the KD subcohort to COVID-19 pediatric controls or adult controls, independently of sex and ethnicity (OR 6.8; 95% CI 1.7–35.1; *p* = 0.009 and OR 9.3; 95% CI 2.6–31.8; *p* = 0.0003), and to controls overall (*n* = 2927) independently of sex, ethnicity, and the presence of COVID-19 (OR 6.8; 95% CI 1.7–35.1; *p* = 0.007) (**Supplementary Table 4B**). No significant impact of the B allele on classical MIS-C phenotype development was observed (**Supplementary Table 4C**).

**Table 2:** ABO blood groups distribution in cases, COVID-19 pediatric controls, and adult controls (**A**) and the impact of blood group B allele on MIS-C overall development (**B** and **C**).

A.																
ABO blood group	MIS-C overall (*n* = 18)	COVID-19 pediatric controls (*n* = 79)	OR*	95% CI*			*p*-value*	Adult controls (*n* = 2,848)		OR°			95% CI°		*p*-value°	
O	27.8	41.8	0.5	0.2–1.6			0.3	39.7		0.6			0.2–1.6		0.3	
A	33.3	41.8	0.7	0.2–2			0.6	44.8		0.6			0.2–1.6		0.3	
B	33.3	11.4	3.8	1.2–12.9			0.03	11.1		4			1.5–10.8		0.01	
AB	5.6	5.1	1.1	0.1–10.5			1	4.4		1.3			0.2–9.6		0.5	
B.																
B allele	MIS-C overall	COVID-19 pediatric controls	OR*	95% CI*	*p*-value*			Adult controls			OR°			95% CI°		*p*-value°
	(*n* = 18)	(*n* = 79)						(*n* = 2,848)								
0/1/2	61/33/6	83.5/15/1.5	2.8	1.03–7.6	0.04			84.5/15/0.5			3.8			1.5–8.5		0.002
C.			Model 1					Model 2								
B allele	MIS-C overall	Controls overall	OR	95% CI		*p*-value		OR	95% CI			*p*-value				
	(*n* = 18)	(*n* = 2927)														
0/1/2	61/33/6	84.5/15/0.5	3.8	1.53–8.44		0.002		2.9	1.04–8.5			0.04				

Values are reported as percentage. A. *MIS-C vs. COVID-19 pediatric controls. °MIS-C vs. adult controls. B. Logistic regression analysis adjusted for sex and ethnicity. * MIS-C vs. COVID-19 pediatric controls °MIS-C vs. adult controls C. Logistic regression analysis in MIS-C vs. controls overall. Model 1: adjusted for sex and ethnicity. Model 2: further adjusted for the presence of COVID-19 disease and blood group A allele.

Although blood group A tended to be more frequent in classical MIS-C compared to KD, no significant impact of the A allele on MIS-C or KD phenotype development was observed (**Supplementary Table 5**).

Similarly, no significant differences were detected for the other analyzed common risk variants, mainly associated with COVID-19 severity, between cases and controls, or between MIS-C and KD phenotypes (**Supplementary Table 6**).

### Rare damaging variants in immune-related genes

**Figure 2:**
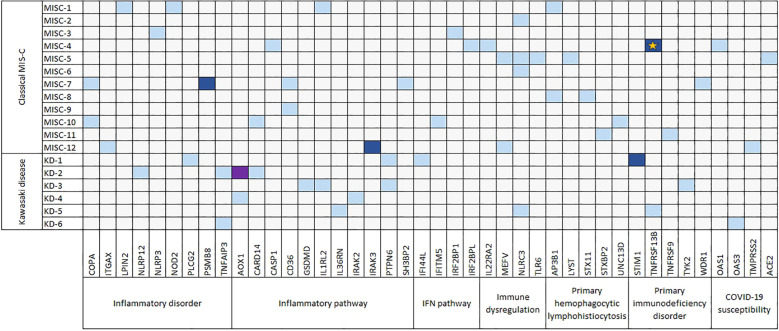
Heatmap-like distribution of rare damaging variants in the MIS-C cohort. Bi-dimensional representation of mutated genes (columns) against individual patients (rows). Patients are arranged by distinct phenotypes: classical MIS-C or Kawasaki disease (KD). Genes are organized by category, and each gene is color-coded based on its mutation frequency and variant type: gray for no variants, light blue for one heterozygous benign/VUS variant, blue for one heterozygous pathogenic variant, and violet for two heterozygous variants. The star highlights the patient with a genetic diagnosis.

Overall, in the MIS-C cohort, we prioritized 49 different rare damaging variants across 39 of the 207 analyzed genes (**Supplementary Table 7**, **Figure 2**). All variants were in heterozygosity; four were classified as P/LP according to ACMG guidelines; the majority (47/49; 96%) were unique to individual patients.

In one child presenting with the MIS-C phenotype, the presence of a pathogenic variant in the *TNFRSF13B* gene allowed the establishment of a diagnosis of autosomal dominant, common variable immunodeficiency (OMIM #240500). The other pathogenic variants were associated with recessive diseases, defining the carrier status.

Only a single variant in the *CD36* gene (p.Gly217Arg) and another one in *PTPN6* (p.Val451Met) were present in two different patients (#MISC-6 and #MISC-7, and #KD-1 and #KD-3, respectively). The gene with the higher mutation rate was *NLCR3*, with four different variants in four different patients. All patients harbored at least one variant in one gene, but the majority (14/18, 78%) harbored variants in more than one gene of the same or different classes. No correlations were found between the presence of one or more rare variants and demographic or clinical data (comorbidity, complications development, coinfections, length of stay, or clinical outcome) (**Supplementary Table 8**). To evaluate whether there was an enrichment of rare variants in the selected genes in patients vs. ethnically matched controls, we compared the total mutational rate. The overall mutation rate was not significantly higher in cases and controls (0.7% vs. 0.6%, *p* = 0.19). Comparing the frequency of single variants, only two variants were nominally more frequent in cases vs. controls (*CD36*, rs200067322, p.Gly217Arg, *p* = 0.01; *PTPN6*, rs62621988, p.Val451Met, *p* = 0.004) (**Supplementary File S1**). Similarly, the fraction of cases with variants in one or more genes tended to be higher, but was not significantly different to that of healthy subjects (subject with one variant: 18/18, 100% cases vs. 228/266, 86% controls; *p* = 0.08; subjects with more than one variant: 14/18, 78% cases vs. 167/266, 63% controls; *p* = 0.2). After stratification according to the clinical phenotype (classical MIS-C vs. KD), no significant differences were observed in the total number of detected variants (33 different variants in 12 MIS-C and 16 different variants in 6 KD). However, in KD, there was a variant enrichment in inflammatory genes (OR 3.9; 95% CI 1.1–14.6; *p* = 0.03 vs. classical MIS-C; OR 4.2; 95% CI 1.5–13.2; *p* = 0.005 vs. controls), while in classical MIS-C, there was a relative enrichment in immune-related genes (OR 4.1; 95% CI 1.1–20.4; *p* = 0.03 vs. KD; OR 1.6; 95% CI 0.8–3.2; *p* = 0.2 vs. controls).

## Discussion

In this study, we characterized the genetic landscape and clinical features of a cohort of Italian children hospitalized with a clinical diagnosis of MIS-C or KD–SARS-CoV-2 (KD) during the first two waves of SARS-CoV-2 infection. Two-thirds of children presented with a classical MIS-C phenotype and the remaining presented with a KD phenotype. In line with literature data, children with a KD phenotype were younger (15, 41) and, although some of them presented with underlying chronic diseases, the median length of hospital stay was shorter.

The main finding was an increased prevalence of blood type B in the cases compared to a cohort of children with COVID-19, hospitalized during the same pandemic period, and to adult local controls representative of the general population. Overall, the presence of group B conferred a 2.9-fold increased risk for MIS-C, independently of sex, ethnicity, and the presence of COVID-19 and blood group A. Stratifying according to classical MIS-C or KD phenotypes, the presence of group B conferred a 6.8-fold increased risk for the KD phenotype as compared to controls overall.

Notably, the ABO locus has been highlighted as the main genetic determinant of SARS-CoV-2 infection susceptibility, with a higher risk of severe infection in individuals carrying the non-O blood groups, particularly in group A (17, 18, 42). A higher frequency of non-O blood group in patients as compared at least to unselected controls is therefore expected to be found, due to heightened susceptibility to being infected after an exposure to SARS-CoV-2. On the other hand, ABO histo-blood type has been linked to risk of several vascular diseases (43), as well as to plasma concentrations of procoagulant proteins, particularly von Willebrand factor (vWF), whose glycation status and stability are determined by the ABO transferase (44, 45). Blood group O has been associated with the lowest vWF levels, whereas individuals homozygous for non-O alleles (namely, BB, AB, and AA genotypes) had the highest levels, followed by group B (BO genotype) and then group A (AO genotype) (46). Notably, children with MIS-C or KD showed prolonged elevations in Factor VIII (FVIII), vWF, and D-dimer levels, reflecting a pro-inflammatory and hypercoagulable state (21, 47). In this context, it could be speculated that the higher prevalence of blood type B, but not type A, in the patient cohort could be related to higher levels of procoagulant and inflammatory proteins, postulating a role of endothelial dysfunction and enhanced activation of the coagulation cascade particularly in the KD phenotype rather than in classical MIS-C pathogenesis. Blood group B has been previously reported to be a risk factor for the formation of coronary artery lesions in patients with KD (48). Based on these data, a role of ABO blood type, and particularly blood type B, could be hypothesized in KD pathogenesis.

To further dissect the genetic predisposition to MIS-C development, we evaluated the presence of rare damaging variants through WES, focalizing the analysis on immune-related genes previously associated with severe COVID-19 or specifically to MIS-C. In one child, a genetic diagnosis of common variable immunodeficiency was established, strengthening the role of immunity alteration in disease pathophysiology. All patients carried rare variants, mainly of unknown significance (VUS), in genes previously associated with MIS-C, although the variants prioritized did not overlap with those reported in previous studies (28, 29, 32–34), likely due to their low frequency and the genetic heterogeneity across ancestries. Moreover, because of the limited sample size, we were not able to demonstrate a significant enrichment in carriage of rare damaging variants in these candidate genes in cases compared to local controls, nor did we find any impact on clinical symptoms or outcomes. Other genetic, epigenetic, or environmental factors could be necessary for disease development.

Although it seemed to be a relative enrichment in variants in immune-related genes in classical MIS-C vs. KD phenotype and in inflammatory genes in KD vs. classical MIS-C, larger sample sizes are needed to capture and validate such differences.

There are some limitations of the present study. The small sample size limited the statistical power to draw definitive conclusions. All the patients were enrolled during the first two pandemic waves, and this limited the ability to evaluate the effects of genetic predisposition to MIS-C development with different SARS-CoV-2 variants. In fact, several studies have showed a significant decline in MIS-C incidence and severity when comparing the ancestral strains and the Delta periods with the Omicron one; moreover, vaccination for COVID-19 has been demonstrated to be protective against the development of MIS-C (7, 10, 49). In addition, we could not measure FVIII or vWF levels in patients, which would have further strengthened the relationship between ABO histo-type and KD phenotype development. Finally, the study cohort was mainly of European ancestry, and because the ABO blood type distribution is related to ethnicity, further studies in larger multiethnic cohorts are needed to confirm the role of the B blood group as a possible risk factor for KD development.

On the other hand, the combined evaluation of rare variants and risk factors associated with MIS-C development and COVID-19 severity for a more comprehensive assessment of the genetic basis of disease pathophysiology is a notable strength. Moreover, we exploited a local control cohort to compare the frequency of variants detected in cases; this allowed us to conclude that some variants previously associated with MIS-C are also frequently observed in healthy individuals and likely do not confer a large increase in the risk of MIS-C.

All in all, our results are consistent with the hypothesis that immune-genetic factors are involved in the pathogenesis of MIS-C, and that endothelial dysfunction and coagulation imbalance may have a key role in KD phenotype pathogenesis and ABO blood type may contribute to disease development.

## Data Availability

The raw data supporting the conclusions of this article will be made available by the authors, without undue reservation.
