# Supplementary material for: Association of blood group B and of rare variants affecting immune system with multisystem inflammatory syndrome in children in an Italian cohort
Source: Front Immunol. 2026 Apr 10;17:1777516. doi: 10.3389/fimmu.2026.1777516 (PMC13106392; doi:10.3389/fimmu.2026.1777516)
Supplement: Supplementary file 1 [file DataSheet1.pdf]

SUPPLEMENTARY

SUPPLEMENTARY TABLES

Supplementary Table S1 page 2
Supplementary Table S2 page 3
Supplementary Table S3 page 8
Supplementary Table S4 page 9
Supplementary Table S5 page 10
Supplementary Table S6 page 11
Supplementary Table S7 page 12
Supplementary Table S8 page 17

SUPPLEMENTARY FIlE

Supplementary File S1 page 18

SUPPLEMENTARY TABLES

Supplementary Table S1. Diagnostic Criteria for classical MIS-C and Kawasaki Disease (KD) subphenotypes.

Diagnostic Criteria for classical MIS-C	Diagnostic Criteria for Kawasaki Disease
- An individual aged <21 years presenting with fever*, laboratory evidence of inflammation**, and evidence of clinically severe illness requiring hospitalization, with multisystem (⩾2) organ involvement (cardiac, renal, respiratory, hematologic, gastrointestinal, dermatologic, or neurological); AND<br>- No alternative plausible diagnoses; AND<br>-Positive for current or recent SARS-CoV-2 infection by RT-PCR, serology, or antigen test; or COVID-19 exposure within the 4 weeks prior to the onset of symptoms.<br><br>*Fever ⩾38.0° C for ⩾24 hours, or report of subjective fever lasting ⩾24 hours<br>**Including, but not limited to, one or more of the following: an elevated C-reactive protein (CRP), erythrocyte sedimentation rate (ESR), fibrinogen, procalcitonin, D-dimer, ferritin, lactic acid dehydrogenase (LDH), or interleukin 6 (IL-6), elevated neutrophils, reduced lymphocytes and low albumin<br><br>Additional Comments:<br>- Some individuals may fulfill full or partial criteria for Kawasaki disease but should be reported if they meet the case definition for MIS-C<br>- Consider MIS-C in any pediatric death with evidence of SARS-CoV-2 infection	Kawasaki Disease:<br>Fever lasting >5 days without other causes with at least 4/5 of the following: - Polymorphous rash - Oral mucous changes, such as injected or fissured lips or strawberry tongue - Cervical lymphadenopathy - Bilateral bulbar conjunctival injection - Peripheral extremity changes, including erythema of palms or soles, periungual desquamation, and edema of hands or feet.<br><br>Incomplete Kawasaki Disease:<br>Fever without any other reason without a sufficient number of clinical diagnostic criteria, associated or not with coronary artery aneurysms.<br><br>Atypical Kawasaki Disease:<br>Fever without any other reason that lasts 3-5 days and is associated with symptoms different from classic clinical features associated or not with coronary artery aneurysms.

Supplementary Table S2. Panel of immune-related genes

Gene	Gene-classes
ABO	COVID-19 susceptibility
ACE2	COVID-19 susceptibility
ACP5	Immune dysregulation
ACTB	Inflammatory pathway
ADA2	Primary immunodeficiency disorder
ADAM17	Inflammatory pathway
ADAR	Inflammatory pathway
AOX1	Inflammatory pathway
AP1S3	Inflammatory pathway
AP3B1	Primary hemophagocytic lymphohistiocytosis (pHLH)
BTNL8	Immune dysregulation
CAPS	Inflammatory pathway
CARD14	Inflammatory pathway
CARD8	Inflammatory pathway
CASP1	Inflammatory pathway
CASP3	Inflammatory pathway
CCR2	Primary immunodeficiency disorder
CD14	Immune dysregulation
CD163	Inflammatory pathway
CD27	Inflammatory pathway
CD36	Inflammatory pathway
CD40	Immune dysregulation
CD84	Immune dysregulation
CECR1	Immune dysregulation
CGAS	IFN pathway
COPA	Inflammatory disorder
CRP	Inflammatory pathway
CSS	Inflammatory pathway
CTLA4	Primary immunodeficiency disorder
CXCL9	Inflammatory pathway
CYBB	Primary immunodeficiency disorder
DOCK8	Primary immunodeficiency disorder
ELANE	Primary immunodeficiency disorder
FCGR2A	Inflammatory pathway
FOXP3	Immune dysregulation
GLI3	Inflammatory pathway
GSDMD	Inflammatory pathway
GZMA	Immune dysregulation
HLA-DOB	Immune dysregulation
IFI16	IFN pathway
IFI27	IFN pathway
IFI27L1	IFN pathway
IFI27L2	IFN pathway
IFI35	IFN pathway
IFI44	IFN pathway

IFI44L	IFN pathway
IFI6	IFN pathway
IFIH1	IFN pathway
IFIT1	IFN pathway
IFIT1B	IFN pathway
IFIT2	IFN pathway
IFIT3	IFN pathway
IFIT5	IFN pathway
IFITM1	IFN pathway
IFITM10	IFN pathway
IFITM2	IFN pathway
IFITM3	IFN pathway
IFITM5	IFN pathway
IFN1	IFN pathway
IFNA1	IFN pathway
IFNA10	IFN pathway
IFNA11P	IFN pathway
IFNA12P	IFN pathway
IFNA13	IFN pathway
IFNA14	IFN pathway
IFNA16	IFN pathway
IFNA17	IFN pathway
IFNA2	IFN pathway
IFNA21	IFN pathway
IFNA22P	IFN pathway
IFNA4	IFN pathway
IFNA5	IFN pathway
IFNA6	IFN pathway
IFNA7	IFN pathway
IFNA8	IFN pathway
IFNAR1	COVID-19 susceptibility
IFNAR2	COVID-19 susceptibility
IFNB1	IFN pathway
IFNE	IFN pathway
IFNG	IFN pathway
IFNGR1	IFN pathway
IFNGR2	IFN pathway
IFNK	IFN pathway
IFNL1	IFN pathway
IFNL2	IFN pathway
IFNL3	IFN pathway
IFNL4	IFN pathway
IFNLR1	IFN pathway
IFNR	IFN pathway
IFRD1	IFN pathway
IFRD2	IFN pathway
IKBKG	Primary immunodeficiency disorder
IL10	Inflammatory pathway

IL17A	Inflammatory pathway
IL18	Inflammatory pathway
IL1A	Inflammatory pathway
IL1B	Inflammatory pathway
IL1RL2	Inflammatory pathway
IL1RN	Inflammatory pathway
IL22RA2	Immune dysregulation
IL36RN	Inflammatory pathway
IL3RA	Inflammatory pathway
IL6	Immune dysregulation
IRAK2	Inflammatory pathway
IRAK3	Inflammatory pathway
IRAK4	Inflammatory pathway
IRF1	IFN pathway
IRF2	IFN pathway
IRF2BP1	IFN pathway
IRF2BP2	IFN pathway
IRF2BPL	IFN pathway
IRF3	COVID-19 susceptibility
IRF4	IFN pathway
IRF5	IFN pathway
IRF6	IFN pathway
IRF7	IFN pathway
IRF8	IFN pathway
IRF9	IFN pathway
ISG15	Primary immunodeficiency disorder
ISG20	IFN pathway
ITGAX	Inflammatory disorder
KLRD1	Immune dysregulation
LPIN2	Inflammatory disorder
LY9	Immune dysregulation
LYST	Primary hemophagocytic lymphohistiocytosis (pHLH)
MEFV	Immune dysregulation
MVK	Primary immunodeficiency disorder
MYBL1	Inflammatory pathway
MYD88	Primary immunodeficiency disorder
NEMO	COVID-19 susceptibility
NFKB1	Inflammatory pathway
NLRC3	Immune dysregulation
NLRC4	Immune dysregulation
NLRP1	Inflammatory disorder
NLRP12	Inflammatory disorder
NLRP2	Inflammatory disorder
NLRP3	Inflammatory disorder
NOD2	Inflammatory disorder
OAS1	COVID-19 susceptibility
OAS2	COVID-19 susceptibility
OAS3	COVID-19 susceptibility

OTULIN	Inflammatory disorder
PAPPA	Inflammatory pathway
PELI1	Immune dysregulation
PIEZO	Inflammatory pathway
PLCG2	Inflammatory disorder
PMAIP1	Immune dysregulation
POLA1	Inflammatory pathway
PRF1	Primary hemophagocytic lymphohistiocytosis (pHLH)
PSMB8	Inflammatory disorder
PSTPIP1	Inflammatory disorder
PSTPIP2	Inflammatory disorder
PTPN18	Inflammatory pathway
PTPN6	Inflammatory pathway
PYCARD	Inflammatory pathway
RAB27A	Immune dysregulation
RAB6A	Immune dysregulation
RIPK1	Inflammatory disorder
RNASEH2A	Immune dysregulation
RNASEH2B	Immune dysregulation
RNASEH2C	Immune dysregulation
RNASEL	IFN pathway
RNF31	Primary immunodeficiency disorder
S100A1	Inflammatory pathway
S100B	Inflammatory pathway
S1PR5	Inflammatory pathway
SAMHD1	Immune dysregulation
SH2D1B	Inflammatory pathway
SH3BP2	Inflammatory pathway
SLC29A3	Immune dysregulation
SLPI	Inflammatory pathway
SOCS1	Primary hemophagocytic lymphohistiocytosis (pHLH)
STAT1	Primary immunodeficiency disorder
STAT2	Primary immunodeficiency disorder
STAT3	Primary immunodeficiency disorder
STIM1	Primary immunodeficiency disorder
STING	Inflammatory disorder
STX11	Primary hemophagocytic lymphohistiocytosis (pHLH)
STXBP2	Primary hemophagocytic lymphohistiocytosis (pHLH)
TBK1	Primary hemophagocytic lymphohistiocytosis (pHLH)
TBX21	Primary immunodeficiency disorder
TGFBR3	Inflammatory pathway
TICAM1	COVID-19 susceptibility
TICAM3	COVID-19 susceptibility
TLR2	Immune dysregulation
TLR3	COVID-19 susceptibility
TLR4	Immune dysregulation
TLR6	Immune dysregulation
TLR7	Immune dysregulation

TLR8	Immune dysregulation
TMPRSS2	COVID-19 susceptibility
TNF	Primary immunodeficiency disorder
TNFAIP3	Inflammatory disorder
TNFRSF13B	Primary immunodeficiency disorder
TNFRSF1A	Immune dysregulation
TNFRSF9	Primary immunodeficiency disorder
TRAF3	Primary immunodeficiency disorder
TREX1	Immune dysregulation
TRIF	COVID-19 susceptibility
TRIM69	Inflammatory pathway
TRNT1	Immune dysregulation
TYK2	Primary immunodeficiency disorder
UNC13D	Primary hemophagocytic lymphohistiocytosis (pHLH)
UNC93B1	COVID-19 susceptibility
USP18	Inflammatory pathway
WDR1	Primary immunodeficiency disorder
XIAP	Primary hemophagocytic lymphohistiocytosis (pHLH)

Supplementary Table S3. Blood test values in the overall cohort and after stratification for clinical subphenotype.

	Overall MIS-C (n=18)	Classical MIS-C (n=12)	KD (n=6)	p-value
Leukocytes (10^3/mL)	13.9 [12.2-22]	16.1 [12.8-22.8]	12.9 [7.9-16.8]	0.16
Platelets (10^3/mL)	587 [489.7-633]	587 [483.2-629]	592.5 [460.2-654]	0.67
CRP (mg/L)	11.6 [8-8-13.2]	11.7 [10.9-13]	5,97 [3.12-14.6]	0.16
ESR (mL/h)*	83 [55-95.5]	69.5 [41.7-87.2]	91 [83.5-101]	0.04
PCT (ng/mL)°	3.9 [1.6-21.4]	8.9 [0.8-32.3]	2.1 [2-2.3]	0.29
ALT (U/L)	33.5 [17-104.2]	85 [31.2-105.7]	18.5 [16.2-29]	0.03
Ferritin (ng/mL)	411 [209-862]	534 [406.2-1108.5]	181 [159.5-251]	0.001
LDH (mU/mL)	313.5 [262-375]	268 [250.2-379.7]	320 [313.7-360.2]	0.18
Pro-BNP (pg/mL)§	4721 [2065-5428]	4737 [3042-6291]	231	0.2
D-Dimer (ng/mL)#	2748 [1823-5520]	2748 [1907-8246.5]	2395 [942.5-5097]	0.33

Data are shown as median [IQR]. CRP: C reactive protein, ESR: erythrocyte sedimentation rate, PCT: procalcitonin, ALT: alanine transaminase; LDH: lactate dehydrogenase; pro-BNP: pro-brain natriuretic peptide. * Available for 13 patients: 8 with MIS-C and 5 with KD; ° Available for 10 patients: 8 with MIS-C and 2 with KD; § Available for 11 patients: 10 with MIS-C and 1 with KD; # Available for 16 patients: 12 with MIS-C and 4 with KD. P values were calculated among pairs through Kruskal-Wallis test for continuous variables (non-normality assumed)

Supplementary Table S4. Impact of blood group B allele on phenotypes (classical MIS-C or KD) development (**A**) and compared to controls (**B** and **C**)

A.

B allele	KD (n=6)	Classical MIS-C (n=12)	OR	95% CI	p value
0/1/2	33/50/17	75/25/0	5.8	0.99-56.1	0.07

B.

B allele	KD (n=6)	COVID-19 pediatric controls (n=79)	OR*	95% CI*	p value*	Adult controls (n=2848)	OR°	95% CI°	p value°	Controls overall (n=2927)	OR§	95% CI§	p value§
0/1/2	33/50/17	83.5/15/1.5	6.8	1.7-35.1	0.009	84.5/15/0.5	9.3	2.6-31.8	0.0003	84.4/15.1/0.5	6.8	1.7-35.1	0.007

C.

B allele	Classical MIS-C (n=12)	COVID-19 pediatric controls (n=79)	OR*	95% CI*	p value*	Adult controls (n=2848)	OR°	95% CI°	p value°
0/1/2	75/25/0	83.5/15/1.5	1.5	0.3-5.5	0.5	84.5/15/0.5	1.8	0.4-5.8	0.4

Values are reported as percentage. **A.** Unadjusted logistic regression model: KD subphenotype vs classical MISC. **B.** Logistic regression analysis adjusted for sex and ethnicity * KD vs COVID-19 pediatric controls. ° KD vs adult controls; § KD vs controls overall further adjusted for the presence of COVID-19 **C.** Logistic regression analysis adjusted for sex and ethnicity * Classical MIS-C vs COVID-19 pediatric controls ° Classical MIS-C vs adult controls

Supplementary Table S5. ABO blood groups distribution in classical MIS-C or KD subphenotype (**A**) and impact of A allele on phenotype development (**B**, **C** and **D**)

A.

ABO blood group	Classical MIS-C (n=12)	KD (n=6)	OR	95% CI	p value
O	33.3	16.7	2.5	0.2-29.2	0.6
A	41.7	16.7	3.6	0.3-40.7	0.6
B	25	49.9	0.3	0.04-2.6	0.3
AB	0	16.7	na	na	0.3

B.

A allele	Classical MIS-C (n=12)	KD (n=6)	OR	95% CI	p value
0/1/2	58.3/16.7/25	66.7/33.3/0	1.8	0.4-12.7	0.5

C.

A allele	KD (n=6)	COVID-19 pediatric controls (n=79)	OR*	95% CI*	p value*	Adult controls (n=2848)	OR°	95% CI°	p value°
0/1/2	66.7/33.3/0	53/42/5	0.5	0.07-2.5	0.4	50.7/41.3/8	0.5	0.07-1.9	0.3

D.

A allele	Classical MIS-C (n=12)	COVID-19 pediatric controls (n=79)	OR*	95% CI*	p value*	Adult controls (n=2848)	OR°	95% CI°	p value°
0/1/2	58.3/16.7/25	53/42/5	1.2	0.4-3	0.7	50.7/41.3/8	1	0.4-2.3	0.9

A. Values are reported as percentage. **B.** Classical MISC vs KD subphenotype at logistic regression model adjusted for sex and ethnicity. **C.** * KD vs COVID-19 pediatric controls at logistic regression model adjusted for sex and ethnicity. ° KD vs adult controls at logistic regression model adjusted for sex and ethnicity. **D.** * Classical MIS-C vs COVID-19 pediatric controls at logistic regression model adjusted for sex and ethnicity. ° Classical MIS-C vs adult controls at logistic regression model adjusted for sex and ethnicity.

Supplementary Table S6. Frequency distribution of common risk variant genotypes for COVID-19 between cases and COVID-19 pediatric controls or adult controls, and between the classical MIS-C and KD subphenotypes.

	Overall MIS-C (n=18)	COVID-19 pediatric controls (n=79)	p value*	Adult controls (n=2848)	p value°	Classical MIS-C (n=12)	KD (n=6)	p value§
OAS1/2/3 rs10735079, allele A	11.1/50/38.9	14.4/42.3/43.3	0.9	14.4/48.3/37.2	0.7	16.7/41.7/41.7	0/66.7/33.3	0.8
DPP9 rs2109069, allele A	50/44.4/5.6	52.6/39.2/8.2	0.9	54.1/38.37.6	0.9	50/41.7/8.3	50/50/0	0.8
IFNAR2 rs2229207, allele C	83.3/16.7/0	85.6/14.4/0	0.7	81.6/16.9/1.4	0.7	91.7/8.3/0	66.7/33.3/0	0.2
LZTFL1 rs11385942, allele GA	83.3/16.7/0	87.6/11.3/1.1	0.7	82.3/16.9/0.8	0.8	66.7/66.7/0	83.3/16.7/0	1
FUT2 rs601338, allele A	33.3/50/16.7	36/49.5/14.5	0.7	29.4/51.2/19.4	0.7	41.7/41.7/16.7	16.7/66.7/16.7	0.5

Data are reported as percentage of patients with 0, 1, or 2 mutated alleles. At logistic regression analysis: *overall MIS-C vs COVID-19 pediatric controls; °overall MIS-C vs adult controls; §: classical MIS-C vs KD subphenotypes.

Supplementary Table S7. Genetic features of rare variants detected in immune-related genes in the MIS-C cohort.

Case ID	Clinical phenotype	Genes	Chromosome coordinates	Transcript	Variant ID	cDNA	Protein effect	gnomAD	CADD score	Zigosity	Protein effect	ACMG classification	Associated disease/pathway	Gene classes
**MISC-1**	MISC	*LPIN2*	chr18-2951126	NM_001375808.2	rs140609636	c.517G>A	p.Ala173Thr	0.0002	0.4	Het	missense	VUS	Majeed_syndrome	Inflammatory disorder
		IL1RL2	chr2-102805582	NM_003854.4	rs746520610	c.105C>A	p.Ser35Arg	0.0003	18	Het	missense	VUS	Cytokine signaling in inflammatory system	Inflammatory pathway
		AP3B1	chr5-77423949	NM_003664.5	rs199908785	c.1873A>T	p.Thr625Ser	0.00008	11	Het	missense	VUS	Hermansky-Pudlak syndrome 2	pHLH
**MISC-2**	MISC	*NLRC3*	chr16-3599772	NM_178844.4	rs376424417	c.2444G>A	p.Ser861Asn	0.0001	21	Het	missense	VUS	NOD-like receptor signaling pathway	Immune dysregulation
**MISC-3**	MISC	*NLRP3*	chr1-247582260	NM_001243133.2	na	c.158C>T	p.Ala55Val	nr	24	Het	missense	VUS	Familial cold inflammatory syndrome 1	Inflammatory disorder
		IRF2BP1	chr19-46387487	NM_015649.3	na	c.1546G>C	p.Val516Leu	nr	28	Het	missense	VUS	Transcriptional regulation of type I interferon	IFN pathway
**MISC-4**	MISC	*TNFRSF13B*	chr17-16855761	NM_012452.3	rs144718007	c.198C>A	p.Cys66Ter	0.00003	36	Het	stop_ gained	P	Immunodeficiency, common variable, 2	PID
		OAS1	chr12-113355369	NM_016816.4	rs201971047	c.902C>T	p.Pro301Leu	0.00007	31	Het	missense	VUS	Immunodeficiency 100 with pulmonary alveolar proteinosis and hypogammaglobulinemia	COVID-19 susceptibility
		CASP1	chr11-104900466	NM_001257118.3	rs139695105	c.788A>G	p.Asn263Ser	0.0009	0.002	Het	missense	VUS	Cytokine signaling in inflammatory system	Inflammatory pathway

		IL22RA2	chr6-137476124	NM_052962.3	na	c.426C>A	p.Ser142Arg	nr	8	Het	missense	VUS	Cytokine signaling in immune system	Immune dysregulation
		IRF2BPL	chr14-77493432	NM_024496.4	rs927676640	c.704T>C	p.Leu235Pro	nr	24	Het	missense	VUS	Interferon regulatory factor binding protein	IFN pathway
**MISC-5**	MISC	*NLRC3*	chr16-3614281	NM_178844.4	rs369562107	c.657C>A	p.Asp266Glu	0.000004	14	Het	missense	VUS	NOD-like receptor signaling pathway	Immune dysregulation
		LYST	chr1-235967862	NM_000081.4	rs767798238	c.3497G>A	p.Arg1166Gl	0.00001	34	Het	missense	VUS	Chediak-Higashi syndrome	pHLH
		TLR6	chr4-38830352	NM_006068.4	rs776197187	c.743G>T	p.Gly248Val	0.0001	0.06	Het	missense	VUS	Toll-like receptor signaling pathway	Immune dysregulation
		MEFV	chr16-3293624	NM_000243.3	na	c.1863A>C	p.Arg621Ser	nr	24	Het	missense	VUS	Familial Mediterranean fever	Immune dysregulation
**MISC-6**	MISC	*NLRC3*	chr16-3613326	NM_178844.4	rs775549170	c.1612G>A	p.Glu585Lys	0.00001	23	Het	missense	VUS	NOD-like receptor signaling pathway	Immune dysregulation
**MISC-7**	MISC	*PSMB8*	chr6-32811743	NM_148919.4	rs757170542	c.31C>T	p.Arg11Ter	0.00002	26	Het	stop_gained	P	Proteasome-associated autoinflammatory syndrome 1	Inflammatory disorder
		COPA	chr1-160278905	NM_004371.4	rs200604416	c.1205C>T	p.Ser402Phe	0.00005	26	Het	missense	VUS	Autoinflammation and autoimmunity, systemic, with immune dysregulation	Inflammatory disorder
		CD36	chr7-80293761	NM_001001548.2	rs200067322	c.649G>A	p.Gly217Arg	0.0003	33	Het	missense	VUS	Triggering of inflammatory signaling pathways	Inflammatory pathway
		WDR1	chr4-10105567	NM_017491.5	rs200525851	c.182T>C	p.Val61Ala	0.004	23	Het	missense	B	Periodic fever, immunodeficiency, and thrombocytopenia syndrome	PID

		SH3BP2	chr4-2831400	NM_001122681.2	na	c.767G>A	p.Arg313Lys	nr	2.6	Het	missense	VUS	Cytokine signaling in inflammatory system	Inflammatory pathway
**MISC-8**	MISC	*STX11*	chr6-144507985	NM_003764.4	rs540150447	c.221C>T	p.Thr74Met	0.0009	26	Het	missense	LB	Hemophagocytic lymphohistiocytosis, familial, 4	pHLH
		AP3B1	chr5-77298786	NM_003664.5	na	c.3225G>T	p.Glu1075Asp	nr	24	Het	missense	VUS	Hermansky-Pudlak syndrome 2	pHLH
**MISC-9**	MISC	*CD36*	chr7-80293761	NM_001001548.2	rs200067322	c.649G>A	p.Gly217Arg	0.0003	33	Het	missense	VUS	Triggering of inflammatory signaling pathways	Inflammatory pathway
**MISC-10**	MISC	*CARD14*	chr17-78172328	NM_001366385.1	rs73429414	c.1789C>T	p.Arg597Trp	0.003	34	Het	missense	B	Cytokine signaling in inflammatory system	Inflammatory pathway
		COPA	chr1-160267211	NM_004371.4	na	c.2185A>G	p.Met738Val	nr	10	Het	missense	VUS	Autoinflammation and autoimmunity, systemic, with immune dysregulation	Inflammatory disorder
		IFITM5	chr11-298590	NM_001025295.3	na	c.310G>A	p.Val104Met	nr	24	Het	missense	VUS	Interferon-induced transmembrane protein	IFN pathway
		UNC13D	chr17-73832901	NM_199242.3	na	c.1154G>T	p.Gly385Val	nr	23	Het	missense	VUS	Hemophagocytic lymphohistiocytosis, familial, 1	pHLH
**MISC-11**	MISC	*TNFRSF9*	chr1-7993252	NM_001561.6	rs780812476	c.649A>G	p.Arg217Gly	0.00002	18	Het	missense	VUS	Immunodeficiency 109 with lymphoproliferation	PID
		STXBP2	chr19-7712629	NM_006949.4	na	c.1715C>T	p.Thr572Ile	nr	28	Het	missense	VUS	Hemophagocytic lymphohistiocytosis, familial, 5	pHLH
**MISC-12**	MISC	*MEFV*	chr16-3304779	NM_000243.3	rs747515115	c.289C>A	p.Gln97Lys	0.00006	9	Het	missense	LB	Familial Mediterranean fever	Immune dysregulation

		TMPRSS2	chr21-42866328	NM_005656.4	rs150389990	c.193G>A	p.Val102Ile	0.0001	0.06	Het	missense	VUS	SARS-CoV-2 entry	COVID-19 susceptibility
		IRAK3	chr12-66603301	NM_007199.3	rs150116809	c.381+1G>T	//	0.0001	19	Het	splice_donor	LP	Cytokine signaling in inflammatory system	Inflammatory pathway
		ITGAX	chr16-31367306	NM_000887.5	na	c.130T>G	p.Tyr44Asp	nr	26	Het	missense	VUS	Susceptibility to systemic lupus erythematosus	Inflammatory disorder
**KD-1**	Kawasaki disease SARS-CoV2-related	*PLCG2*	chr16-81939053	NM_002661.5	na	c.1408G>A	p.Asp470Asn	0.000004	18	Het	missense	VUS	Autoinflammation, antibody deficiency, and immune dysregulation syndrome	Inflammatory disorder
		IFI44L	chr1-79093863	NM_006820.4	rs771610857	c.263A>C	p.Gln88Pro	0.000004	1	Het	missense	VUS	Interferon-induced protein	IFN pathway
		PTPN6	chr12-7067226	NM_002831.6	rs62621988	c.1351G>A	p.Val451Met	0.0008	32	Het	missense	VUS	Cytokine signaling in inflammatory system	Inflammatory pathway
		STIM1	chr11-3988790	NM_001382567.1	na	c.148C>T	p.Arg50Ter	nr	38	Het	stop_gained	LP	Immunodeficiency	PID
**KD-2**	Kawasaki disease SARS-CoV2-related	*AOX1*	chr2-201459997	NM_001159.4	rs754596150	c.106C>T	p.Arg36Ter	0.00002	28	Het	stop_gained	VUS	Regulation of inflammatory responses	Inflammatory pathway
		NLRP12	chr19-54313268	NM_144687.4	rs761571455	c.1645G>A	p.Ala549Thr	0.000008	13	Het	missense	VUS	Familial cold autoinflammatory syndrome 2	Inflammatory disorder
		TNFAIP3	chr6-138202356	NM_001270508.2	rs377038705	c.2273C>A	p.Pro758His	0.000008	31	Het	missense	VUS	Autoinflammatory syndrome, familial, Behcet-like 1	Inflammatory disorder
		AOX1	chr2-201515755	NM_001159.4	rs138780561	c.2906A>G	p.Asn969Ser	0.0002	10	Het	missense	LB	Regulation of inflammatory responses	Inflammatory pathway
**KD-3**	Kawasaki disease SARS-	*IL1RL2*	chr2-102851594	NM_003854.4	rs199504408	c.1535G>A	p.Arg512Gln	0.00003	13	Het	missense	VUS	Cytokine signaling in inflammatory system	Inflammatory pathway

	CoV2- related	*PTPN6*	chr12-7067226	NM_002831.6	rs62621988	c.1351G>A	p.Val451Met	0.0008	32	Het	missense	VUS	Cytokine signaling in inflammatory system	Inflammatory pathway
		GSDMD	chr8-144644451	NM_024736.7	na	c.1069G>T	p.Val357Leu	nr	8	Het	missense	VUS	Cytokine signaling in inflammatory system	Inflammatory pathway
		TYK2	chr19-10476458	NM_003331.5	na	c.746G>A	p.Arg249Gln	nr	5	Het	missense	VUS	Immunodeficiency 35	PID
**KD-4**	Kawasaki disease SARS-CoV2-related	*AOX1*	chr2-201503018	NM_001159.4	rs112951726	c.2561A>G	p.Lys854Arg	0.001	21	Het	missense	VUS	Regulation of inflammatory responses	Inflammatory pathway
**KD-5**	Kawasaki disease SARS-CoV2-related	*IL36RN*	chr2-113819785	NM_012275.3	na	c.200G>T	p.Cys67Phe	0.000008	28	Het	missense	VUS	Cytokine signaling in inflammatory system	Inflammatory pathway
		NLRC3	chr16-3614136	NM_178844.4	rs201264477	c.802C>T	p.Arg315Cys	0.0005	28	Het	missense	VUS	NOD-like receptor signaling pathway	Immune dysregulation
**KD-6**	Kawasaki disease SARS-CoV2-related	*OAS3*	chr12-113403585	NM_006187.4	rs760359985	c.2440C>T	p.Arg814Cys	0.000004	28	Het	missense	VUS	IFN-pathway	COVID-19 susceptibility
		TNFAIP3	chr6-138202365	NM_001270508.2	rs368859219	c.2282G>A	p.Arg761His	0.00007	25	Het	missense	VUS	Autoinflammatory syndrome, familial, Behcet-like 1	Inflammatory disorder

Abbreviations: Het_heterozygous; P: pathogenic; LP: likely pathogenic; VUS: variant of uncertain significance; LB: likely benign; B. benign; pHLH: primary hemophagocytic lymphohistiocytosis; PID: primary immunodeficiency disorder

Supplementary Table S8: Demographics and clinical data in patients with single vs. multiple rare variants in immune-related genes

	Single variant (n=4)	Multiple variants (n=14)	p-value
Sex, male	3 (30)	7 (70)	0.5
Age, years	9 [3.7-11.2]	7 [2-9.2]	0.4
Comorbidity, yes	0 (0)	2 (14.3)	1
Complications, yes	2 (50)	9 (64.3)	1
shock	0	1 (7.4)	1
cardiac dysfunction	2 (50)	9 (64.3)	1
pneumonia	1 (25)	4 (28.6)	1
gastroenteric complications	2 (50)	7 (50)	1
Coinfections, yes	2 (50)	5 (41.7)	1
Lenght of stay, days	11.5 [9-16.2]	12 [7.7-15]	0.8
Sequelae, yes	0	0	na
Clinical phenotype, MISC	3 (75)	9 (64.3)	1

Data are shown as N (%), or median [IQR], when appropriate. P values were calculated among pairs through Kruskal-Wallis test for continuous variables (non-normality assumed) and Fisher test for categorical variables

SUPPLEMENTARY FIlE

Supplementary File S1. Comparison of the frequency of detected variants in cases and controls.

In bold the variants nominally more frequent in cases vs controls; p-value was calculated using Fisher's exact test.
